# Mycoflora and Co-Occurrence of Fumonisins and Aflatoxins in Freshly Harvested Corn in Different Regions of Brazil

**DOI:** 10.3390/ijms10115090

**Published:** 2009-11-24

**Authors:** Liliana O. Rocha, Viviane K. Nakai, Raquel Braghini, Tatiana A. Reis, Estela Kobashigawa, Benedito Corrêa

**Affiliations:** 1 Department of Microbiology, Institute of Biomedical Sciences, University of São Paulo, São Paulo, SP, Brazil; E-Mails: lilianarocha@usp.br (L.O.R.); vivinakai@hotmail.com (V.K.N.); raquelbraghini@yahoo.com.br (R.B.); tareis@usp.br (T.A.R.); 2 Department of Food Engineering, School of Animal Science and Food Engineering, University of São Paulo, Pirassununga, SP, Brazil; E-Mail: estelakop@hotmail.com (E.K.)

**Keywords:** fumonisins, aflatoxins, corn (*Zea mays* L.), mycotoxins, mycoflora, Fusarium verticillioides, Aspergillus flavus

## Abstract

Natural mycoflora and co-occurrence of fumonisins (FB_1_, FB_2_) and aflatoxins (AFB_1_, AFB_2_, AFG_1_ and AFG_2_) in freshly harvested corn grain samples from four regions of Brazil were investigated. *Fusarium verticillioides* was predominant in all samples. Analysis of fumonisins showed that 98% of the samples were contaminated with FB_1_ and 74.5% with FB_1_ + FB_2_, with toxin levels ranging from 0.015 to 9.67 μg/g for FB_1_ and from 0.015 to 3.16 μg/g for FB_2_. Twenty-one (10.5%) samples were contaminated with AFB_1_, seven (3.5%) with AFB_2_ and only one (0.5%) with AFG_1_ and AFG_2_ Co-contamination with aflatoxins and fumonisins was observed in 7% of the samples. The highest contamination of fumonisins and aflatoxins was observed in Nova Odessa (SP) and Várzea Grande (MT), respectively. The lowest contamination of these mycotoxins was found in Várzea Grande and Nova Odessa, respectively.

## 1. Introduction

Corn (*Zea mays* L.) is grown in hot and temperate regions around the world and is the second most cultivated crop in Brazil. The country is currently the world’s third largest producer after the United States and China, with an average production of about 43 million tons over the last five years [1]. Since corn grain posses a high nutritional value, it is used for the preparation of diverse food products, and represents a relevant and important socioeconomic factor in many regions of the world [2].

Corn can be affected by different toxigenic fungi, especially *Fusarium verticillioides* (Sacc.) Nirenberg (=*F. moniliforme* Sheldon) and *F. proliferatum* (Matsushima) Nirenberg and *Aspergillus flavus* Link, the main producers of fumonisins and aflatoxins, respectively. Contamination with these toxins is one of the main factors compromising the quality of corn products [3].

Twenty-eight fumonisin analogs have been described so far, with fumonisin B_1_ (FB_1_) being the most important of the group due to its abundance in corn grain and because it is the most toxic among the fumonisin isomers [4]. FB_1_ has been shown to be hepatotoxic to all animals species studied so far [5–9]. In addition, FB_1_ is known to cause leukoencephalomalacia in horses [6] and pulmonary edema and hydrothorax in swine, and to exert nephrotoxic as well as hepatotoxic activity in rats [8] and rabbits [10–12]. In humans, FB_1_ has been associated with esophageal cancer [4]. On the basis of toxicological evidence, the International Agency for Research on Cancer (IARC) has established that FB_1_ is potentially carcinogenic (class 2B) to humans [13].

Aflatoxins are secondary metabolites produced by toxigenic strains of *A. flavus* and *A. parasiticus*. Chemically, aflatoxins belong to the bifuranocoumarin group, with aflatoxins B_1_ (AFB_1_), B_2_ (AFB_2_), G_1_ (AFG_1_) and G_2_ (AFG_2_) being the most toxic. Liver is the main organ affected by these toxins. In addition to its hepatotoxic action, AFB_1_ is also highly mutagenic, carcinogenic and probably teratogenic to animals. The IARC classifies AFB_1_ within class 1 of human carcinogens [14].

In Brazil, the presence of aflatoxins in corn is regulated by the Ministry of Agriculture through Decree 183 of March 21, 1996 and Resolution 274 of October 15, 2002 of the National Sanitary Surveillance Agency, which establish a maximum limit of 20 μg/kg for the sum of aflatoxins B_1_, B_2_,G_1_ and G_2_ [15,16]. No limit has been established yet in Brazil for fumonisins in foods. However, the countries of European Union recommend a limit of 2,000 μg/kg for the sum of FB_1_ and FB_2_ in unprocessed corn [17]. In addition, Food and Drug Administration (FDA) recommends levels of 2 to 4 ppm for products intended for human consumption and of 5 to 100 ppm for products destined for animal feeding [18].

Occurrence of fumonisins and aflatoxins in corn and corn products has been a world-wide problem. In view of the difficulty in removing these mycotoxins, monitoring of grains during the period from planting to harvest is important for the control of exposure to these toxins [19]. Therefore, the objective of the present study was to evaluate the occurrence of fungi and the incidence of fumonisins and aflatoxins in corn grains freshly harvested in four regions in Brazil.

## 2. Experimental Section

### 2.1. Corn samples

A total of 200 corn samples (Agromen 2012 hybrid, AGN 2012) freshly harvested from the following four different regions of Brazil were analyzed: Várzea Grande (Mato Grosso, MT); Nova Odessa (São Paulo, SP); Santa Maria (Rio Grande do Sul, RS), and Oliveira do Campinhos (Recôncavo Bahiano, Bahia, BA). The study regions are characterized by variations in climatic conditions during the period from sowing to harvest (Table 1). Samples (50 per region) were sown in November 2004 and harvested in April 2005. Sampling was performed according to the method proposed by Delp *et al*. [20]. The area selected for sowing in each region was divided into 10 parcels of 80 m^2^ each. Five parcels were chosen from each area, and 10 ears of corn were sampled from each parcel. In this way, five samples were collected at each region, containing 10 ears of corn. A 1 kg subsample was taken from each of these samples and assayed for mycoflora, aflatoxin and fumonisins contents, and water activity (*a_w_*).

### 2.2. Water activity

The water activity (*a_w_*) was determined with an Aqualab CX-2 apparatus (Decagon Devices, Inc., Pullman, WA, USA).

### 2.3. Isolation, enumeration and identification of the mycoflora

Approximately 30 g of grains were obtained from each corn subsample (1 kg) and disinfected with 0.4% sodium hypochlorite solution for 2 min, followed by washing with sterile distilled water for elimination of external contaminants. After disinfection, some grains were randomly separated and directly seeded into Petri dishes containing Dichloran Rose Bengal Chloramphenicol agar (DRBC). Three plates containing 11 grains were used for each sample. The plates were incubated at 25 °C for five days and the results were expressed as the percentage of total grains infected with fungi [21].

Fungal colonies were identified to the genus level and those belonging to the genera *Fusarium* and *Aspergillus* were identified to the species level according to Raper and Fennell [22], Pitt and Hocking [23], Nelson *et al*. [24] and Leslie and Summerell [25].

### 2.4. Determination of fumonisins

Twenty grams of each previously triturated sample was transferred to a 250 mL centrifuge bottle and 50 mL of a mixture of acetonitrile-methanol-water (25:25:50, v/v/v) was added. After shaking for 20 min in a horizontal mechanical shaker, the samples were centrifuged at 2,500 × g for 10 min and the supernatant was filtered through Whatman No. 4 filter paper (12 cm). The steps were repeated for the precipitate and the filtrates were collected. Ten milliliter was combined with 40 mL phosphate buffer saline (PBS), pH 7.0 (8.0 g NaCl, 1.2 g anhydrous Na_2_HPO_4_, 0.2 g KH_2_PO_4_, 0.2 g KCl in approximately 990 mL water, pH was adjusted with 2M HCl, and diluted to 1 L) and the mixture was shaken. Diluted extracts were filtered through microfiber filter paper (Whatman GF/A, 9 cm) and 10 mL was collected for purification on an immunoaffinity column (FumoniTest - Vicam) at a flow rate of 1–2 drops/s. The column was washed with 10 mL PBS (pH 7.0) at 1–2 drops/s for removal of residues. Fumonisins were eluted with 1.5 mL methanol (HPLC grade) at 1 drop/s, evaporated to residue in a water bath, and submitted to detection and quantification [26].

The residue was resuspended in 200 μL acetonitrile:water (50:50, v/v). After derivatization of 50 μL of the sample extract diluted in 50 μL *ortho*-phthaldehyde (OPA) solution (40 mg OPA, 1 mL methanol, 5 mL 0.1 M sodium tetraborate and 50 μL 2-mercaptoethanol) for 2 min, fumonisins were injected into a Shimadzu liquid chromatograph (model LC-10AD) equipped with an injector and 20-μL fixed loop (Rheodyne). After separation on a C-18 reverse phase column (5 ODS-20, 150 × 4.6 mm, Phenomenex), fumonisins were detected using a fluorescence detector (model RF-10AXL) at excitation and emission wavelengths of 335 and 440 nm, respectively. Acetonitrile:water:acetic acid (480:520:5, v/v/v) was used as mobile phase at a flow rate of 1.0 mL/min. Column temperature was 30 °C and room temperature 22–23 °C. Under these conditions, retention time was 9 and 20 min for FB_1_ and FB_2_, respectively [26].

Fumonisins were quantified based on a calibration curve using standard solutions of FB_1_ and FB_2_. Concentrations of standard solutions ranging from 0.025 to 2000 ng/μL for FB_1_ and from 0.0125 to 1000 ng/μL for FB_2_ were used. Coefficient of correlation was 0.99277 for FB_1_ and 0.995047 for FB_2_.

Quantification limit for FB_1_ was 0.015 μg/g, with a mean recovery of 92.38% and standard deviation of 13.68% (five replicates). For FB_2_, quantification limit was 0.015 μg/g, with a mean recovery of 85.39% (five replicates, standard deviation: 6.87%).

### 2.5. Determination of aflatoxins

Aflatoxins B_1_ and B_2_ were determined according to the method described by Soares and Rodriguez-Amaya [27]. Briefly, 50 g of each sample of grains was extracted with 270 mL methanol and 30 mL 4% potassium chloride. Samples were blended at moderate speed for 30 min and filtered, and 150 mL of the filtrate was collected into a graduated cylinder. Next, 150 mL 30% ammonium sulfate solution and 50 mL diatomaceous earth were added. The suspension was filtered through filter paper and a 150-mL aliquot was transferred to a separation funnel containing 150 mL distilled water. The toxins were extracted three times with 10 mL chloroform. The chloroform extracts were collected in a beaker and the solvent was evaporated in a water bath at 60 °C.

The dried extracts were resuspended in 500 μL chloroform and immediately subjected to thin-layer chromatography. Final identification and quantification of aflatoxins were performed by one-dimensional thin-layer chromatography on precoated silica gel plates G-60 (Merck). The plates were developed in a saturated chamber with chloroform-acetone (9:1, v/v). The fluorescent spots corresponding to AFB_1_ and AFB_2_ were observed in a dark chamber under ultraviolet light (λ = 366 nm). Aflatoxins were determined by visual comparison with AFB_1_ and AFB_2_ standards prepared. Confirmatory tests were carried out using trifluoroacetic acid [28]. The quantification limit of the method was 2 μg/kg for AFB_1_, with a mean recovery of 91.99% and standard deviation of 6.93% (five replicates), and 4 μg/kg for AFB_2_, with a mean recovery of 92% and standard deviation of 13.32% (five replicates).

### 2.6. Statistical analysis

Results were analyzed statistically using the Gamlss R 2.8.1 package and Statistical Analysis Software (SAS) version 8.0. Spearman’s correlation coefficient was used to analyze fungal growth in the grains. Gamlss model (5% level of significance) was used to determine the effect of different regions studied and water activity on fungal growth, as well as the effect of the different regions on incidence of mycotoxins [29]. Gamlss model (5% level of significance) was also used to evaluate the effect of different regions and fungal growth on incidence of mycotoxins. Bonferroni’s test was used for multiple comparisons between regions [30]. Low frequency of contamination with AFB_2_, AFG_1_ and AFG_2_ did not permit model fit for inferential analysis of these toxins.

## 3. Results and Discussion

The results regarding the mycoflora detected in corn grain samples obtained from four different regions (São Paulo, Mato Grosso, Rio Grande do Sul and Bahia) are shown in Table 2. Fungal contamination was observed in 100% of the corn samples analyzed, with *Fusarium verticillioides* being the most frequent species in all regions.

The following species and genera were isolated from the 50 corn grain samples collected in Nova Odessa-SP: *Fusarium verticillioides* (88.6%), *F. proliferatum* (7.6%), *Penicillium* (15.4%), *Cladosporium* (10.4%), *Trichoderma* (1.1%), and *Mucor* (0.1%). *A_w_* values ranged from 0.78 to 0.89, with a mean of 0.87.

The following fungi were detected in samples from Santa Maria-RS: *F. verticillioides* (86.7%), *F. proliferatum* (4.6%), *Penicillium* (19.2%), *Aspergillus flavus* (9.8%), *Cladosporium* (1.6%), *Trichoderma* (1.3%), and non-sporulating fungi (0.1%). *A_w_* values ranged from 0.74 to 0.85, with a mean of 0.82.

The following species and genera were isolated in Várzea Grande-MT: *F. verticillioides* (84.2%), *F. proliferatum* (3.2%), *Aspergillus flavus* (13.8%), *A. niger* (0.2%), *Cladosporium* (0.8%), *Penicillium* (0.87%), and *Curvularia* (0.2%). *A_w_* values ranged from 0.71 to 0.81 in the corn samples analyzed, with a mean of 0.76.

The following fungi were detected in the 50 corn samples collected in Oliveira dos Campinhos - BA: *F. verticillioides* (84.9%), *F. subglutinans* (3.9%), *F. anthophilum* (1.6%), *Aspergillus flavus* (12.0%), *A. niger* (0.6%), *Penicillium* (1.9%), *Mucor* (1.6%), *Rhizopus* (1.1%), *Cladosporium* (0.4%), and *Neurospora* (0.3%). *A_w_* values ranged from 0.76 to 0.87, with a mean of 0.83.

Spearman’s correlation test revealed a moderately high, negative correlation (*r =* −0.61; *p* < 0.0001) between isolation of genera *Fusarium* and *Aspergillus*, suggesting that samples with a high percentage of *Fusarium* contamination tend to have low contamination with *Aspergillus*.

Inferential analysis of the growth of *Fusarium* was performed using the Gamlss model with beta inflated distribution [29]. Results showed that frequency of *Fusarium* in corn grains varied as function of *a_w_* and of the different regions studied (*p* < 0.0001).

The same model was used for analysis of growth of *Aspergillus*, but frequency of *Fusarium* was included as a predictive variable. *Aspergillus* growth varied as function of growth of *Fusarium* (*p* = 0.0002), as well as function of *a_w_* and region (*p* < 0.0001).

According to Lillehoj *et al*. [31] and Deacon [32], *F. verticillioides* is a strong competitor of *A. flavus*. These fungi present a passive antagonistic relationship in which growth is inhibited by competition for space or essential nutrients, with advantages for microorganisms that are present in larger number or are better adapted to the substrate. Marín *et al*. [33], studying the influence of water activity and temperature on interaction between filamentous fungi, observed that *F. verticillioides* strains became more competitive and were able to inhibit other fungal genera such as *Aspergillus* and *Penicillium* when maintained on a substrate with a high *a_w_* at a temperature of 15 °C.

However, according to Cuero *et al*. [34], there is no indication of antagonism with *A. flavus* or of any effect on the levels of aflatoxin in maize grains. There could be a synergism between these two species, therefore, *F. verticillioides* can stimulate the metabolism of *A. flavus* and increase aflatoxin production. In the present study, growth of *Fusarium* spp. was probably favored in the region where mean *a_w_* levels were around 0.87 (Nova Odessa-SP). On the other hand, the lowest *a_w_* (0.76) observed in Várzea Grande-MT resulted in a higher frequency of isolation of *Aspergillus* spp. (14%) and a lower frequency of *Fusarium* spp. (84.2%) (Table 2).

Fumonisins were the most frequent mycotoxins in 200 samples analyzed. This result agrees with those obtained by mycological analysis demonstrating that *F. verticillioides* was the predominant species. Contamination with FB_1_ was observed in 98% of freshly harvested corn analyzed (196 samples) and contamination with FB_1_ + FB_2_ in 74.5% (149 samples). Frequency of contamination and concentrations of FB_1_ and FB_2_ are shown in Table 3.

Our results showed that 57.7% (86 samples) of the 149 samples contaminated with FB_1_ + FB_2_ had levels higher than those recommended by the European Union [17] (2 μg/g) and 28.9% (43 samples) had levels higher than 4 μg/g, the maximum level recommended by the FDA [18] for products intended for human consumption.

Highest levels of fumonisin contamination were observed in Nova Odessa-SP, with FB_1_ concentrations ranging from 0.091 to 9.67 μg/g (mean: 2.81 μg/g) and FB_2_ concentrations from 0.017 to 3.06 μg/g (mean: 0.95 μg/g). In this region, the meteorological data (mean temperature: 24.6 °C; relative humidity: 79.3% and rainfall index: 6 mm) might have favored the production of these toxins.

According to Hennigen *et al*. [35], elevated fumonisin levels in corn are associated with high relative air humidity. Gong *et al*. [36] reported that temperature, relative humidity and rainfall were responsible for high levels of contamination of corn grains with FB_1_ in China. According to FDA [18], these meteorological parameters in different geographic regions during pre-harvest and harvest periods were found to highly influence on fumonisin levels in corn.

In Brazil, studies of the occurrence of FB_1_ and FB_2_ in freshly harvested corn have revealed detection rates of 92.3% and 81%, respectively. Most of these samples collected in different regions contained levels ranging from 0.02 to 78.92 μg/g for FB_1_ (mean: 4.9 μg/g) and from 0.02 to 29.16 μg/g for FB_2_ (mean: 3.9 μg/g) [19,37–47]. In the present study, presence of FB_1_ ranged from 92% to 100%, toxin levels from 0.015 to 9.67 μg/g, and mean concentration from 0.72 to 2.81 μg/g. Presence of FB_2_ ranged from 50% to 98%, toxin levels from 0.02 to 3.16 μg/g and mean concentration from 0.15 to 0.95 μg/g (Table 3). It should be emphasized that this is the first report of occurrence of fumonisins in corn from Bahia and the first comparative study between corn-producing regions in Brazil.

Gamlss model with a gamma distribution was used for statistical analysis of FB_1_ since samples had a low probability of being equal to zero [49]. Mean contamination of grain with FB_1_ varied as function of region (*p* < 0.0001) and growth of *Fusarium* (*p* = 0.0009). Since contamination with FB_2_ assumed values ≥ 0, with a high probability of being equal to zero, Gamlss model with a zero adjusted inverse Gaussian distribution was used for analysis [48]. Probability of fumonisin level being greater than or equal to 0 is 100% since contamination level cannot be a negative value (*p* < 0.0001 and *p* = 0.0206, respectively) and it varied according to region. Using these models, the average probability of contamination of corn grains with FB_1_ and FB_2_ was higher in Nova Odessa-SP and Oliveira dos Campinhos-BA than in Várzea Grande-MT and Santa Maria-RS.

Among 200 samples analyzed, 21 (10.5%) were contaminated with AFB_1_, seven (3.5%) with AFB_2_ (Table 4) and only one (0.5%) with AFG_1_ and AFG_2_. Sixteen (76.2%) of 21 positive samples had total aflatoxins concentration (B_1_ + B_2_ + G_1_ + G_2_) higher than the limit established by Brazilian regulations (20 μg/kg) [15,16].

In Nova Odessa (SP), only one sample (2%) was contaminated with AFB_1_ at a concentration of 34.2 μg/kg (mean: 0.68 μg/kg). This finding might be explained by the absence of *A. flavus* in corn collected in this region. Pozzi *et al*. [50], analyzing 130 corn samples from Ribeirão Preto, SP, also reported low contamination with AFB_1_. Almeida *et al*. [42], investigating the occurrence of aflatoxins in corn from Capão Bonito and Ribeirão Preto, did not observe contamination with this toxin in any of the 57 samples. According to Salay and Mercadante [51], percentage of contamination of corn and derivatives with aflatoxins in the State of São Paulo (11.5%) is lower than that observed in North (32.9%), South (33.4%) and Northeast (96.9%) regions of Brazil.

In Santa Maria (RS), seven (14%) samples were contaminated with AFB_1_ and five (10%) with AFB_2_ at levels ranging from 13.7 to 1,393 μg/kg (mean: 50.5 μg/kg) and from 5.6 to 55.7 μg/kg (mean: 3.5 μg/kg), respectively. One sample was contaminated with AFG_1_ (39.2 μg/kg, mean: 0.78 μg/kg) and AFG_2_ (29.7 μg/kg, mean: 0.59 μg/kg). In Várzea Grande (MT), nine (18%) samples were contaminated with AFB_1_ and two (4%) with AFB_2_ at levels ranging from 6.8 to 976.1 μg/kg (mean: 27.7 μg/kg) and from 22.3 to 33.4 μg/kg (mean: 1.11 μg/kg), respectively. Among samples collected in Oliveira dos Campinhos (BA), four (8%) were contaminated with AFB_1_ at levels ranging from 13.7 to 47.8 μg/kg (mean: 2.05 μg/kg). Aflatoxin B_2_ was not detected in samples from this region.

Gamlss model with a zero-adjusted Gaussian distribution was used for inferential analysis of AFB_1_ contamination. The probability of AFB_1_ contamination being greater than or equal to 0 is 100%, since contamination level cannot be less than 0 (*p* = 0.0030 and *p* = 0.0006, respectively) and it varied only as a function of frequency of *A. flavus*.Thus, the higher the fungus frequency, the higher the probability of contamination of corn with AFB_1_.

Some Brazilian studies have reported occurrence of aflatoxins in corn grains. In these studies, the frequency of positive samples ranged from 11.3% to 77.1% (mean: 30.6%), with toxin levels ranging from 1 to 1906 μg/kg [41,43,52–56]. In the present investigation, the frequency of positive samples for AFB_1_ and AFB_2_ ranged from 2% to 18%, and toxin levels ranged from 5.6 to 1393 μg/kg (mean: 2.05 to 50.5 μg/kg) (Table 4).

According to Lacey *et al*. [57], temperatures and *a_w_* for growth of *A. flavus* range from 6 °C to 45 °C and from 0.78 to 0.80, respectively. Marín *et al*. [33] demonstrated that *Aspergillus* species are more competitive at high temperatures, with optimum growth rate for *A. niger* and *A. flavus* ranging from 30 °C to 37 °C. Zorzete *et al*. [58], analyzing the distribution of aflatoxins and fumonisins in corn kernels inoculated with *A. flavus* and *F. verticillioides* from flowering to harvest, observed that *A. flavus* was better adapted to high temperature and low humidity, with the demonstration of a significant negative linear correlation between fungal frequency and rainfall.

## 4. Conclusions

In the present study, better adaptation of *F. verticillioides* to the temperature water activity levels, contributed to high frequency of isolation of this species and lower incidence of *A. flavus* in corn samples collected from diverse locations in Brazil. Only 7% of 200 corn samples collected in four regions were contaminated with both aflatoxins and fumonisins. However, frequency of samples testing positive for fumonisins as well as high frequency of isolation of *Fusarium* spp., demonstrate the need for effective prevention and control strategies in order to reduce risks to human and animal health.

## Figures and Tables

**Table 1.** Meteorological data from sowing to harvest (November, 2004 to March, 2005) of four regions in Brazil.

**Location**	**Mean temperature (**°**C)**	**Standard deviation**	**Mean relative humidity (%)**	**Standard deviation**	**Mean rainfall (mm)**	**Standard deviation**
**Várzea Grande (MT)**	27.1	2.1	40.0	26.4	3.4	6.2
**Santa Maria (RS)**	22.5	3.5	71.3	11.8	2.8	9.0
**Oliveira dos Campinhos (BA)**	28.0	1.3	73.7	9.3	6.1	18.0
**Nova Odessa (SP)**	24.6	2.1	79.3	7.7	6.0	13.4

**Table 2.** Mean relative frequency of fungi isolated from 200 corn samples in four regions of Brazil.

**Sampling region**	***a_W_^C^***	**Frequency of isolated fungi (%)**									
		*Fusarium* spp.	*Aspergillus* spp.	*Cladosporium* spp.	*Trichoderma* spp.	*Mucor*spp.	*Penicillium* spp.	NSF *e*	*Curvularia* spp.	*Rhizopus* spp.	*Neurospora* spp.
**MT*a, b***	0.76	87.4	14	0.8	ND	ND	0.8	ND	0.2	ND	ND
**RS*a, b***	0.82	91.3	9.8	1.6	1.3	ND	19.2	0.1	ND	ND	ND
**BA*a, b***	0.83	90.4	12.6	0.4	ND	1.6	1.9	ND	ND	1.1	0.3
**SP*a, b***	0.87	96.2	ND *d*	10.4	1.1	0.1	15.4	ND	ND	ND	ND

a Fifty freshly-harvest corn samples from each region were evaluated.

b Regions: Várzea Grande - MT; Santa Maria - RS; Oliveira dos Campinhos - BA; Nova Odessa - SP.

c Mean water activity.

d Not detected.

e Non-sporulating fungus.

**Table 3.** Fumonisins contamination in 200 freshly-harvested corn samples from four regions of Brazil.

**Sampling region**	**Fumonisin B_1_**				**Fumonisin B_2_**			

	**Positive samples (%)**	**Range (**μ**g/g)**	**Mean (**μ**g/g)**	**Standard deviation**	**Positive samples (%)**	**Range (**μ**g/g)**	**Mean (**μ**g/g)**	**Standard deviation**
**MT*a, b***	92	0.015–8.44	0.73	1.4	56	0.02 – 3.03	0.19	0.5
**RS*a, b***	100	0.015–6.27	0.72	1.7	50	0.015–1.18	0.15	0.3
**BA*a, b***	100	0.015–9.42	2.75	1.6	98	0.12–1.31	0.62	0.3
**SP*a, b***	100	0.091–9.67	2.81	1.8	94	0.017–3.16	0.95	0.8

Quantification limit for FB_1_ and FB_2_: 0.015 μg/g for both mycotoxins.

a Fifty freshly-harvest corn samples were evaluated from each region.

b Regions: Várzea Grande-MT; Santa Maria-RS; Oliveira dos Campinhos-BA; Nova Odessa-SP.

**Table 4.** Aflatoxins B_1_ and B_2_ contamination in corn samples from four regions of Brazil.

**Sampling region**	**Aflatoxin B_1_**				Aflatoxin B**_2_**			

	**Positive samples (%)**	**Range (**μ**g/kg)**	**Mean (**μ**g/kg)**	**Standard deviation**	**Positive samples (%)**	**Range (**μ**g/kg)**	**Mean (**μ**g/kg)**	**Standard deviation**
**MT*a, b***	18	6.8–976.1	27.7	139.0	4	22.3–33.4	1.11	5.6
**RS*a, b***	14	13.7–1393.0	50.5	237.9	10	5.6–55.7	3.5	12.2
**BA*a, b***	8	13.7–47.8	2.05	8.0	ND *d*	ND	ND	ND
**SP*a, b***	2*c*	ND–34.2	0.68	4.8	ND	ND	ND	ND

Quantification limit for AFB_1_ and AFB_2_: 2 μg/kg and 4 μg/kg, respectively.

a Fifty freshly-harvest corn samples were evaluated from each region.

b Regions: Várzea Grande - MT; Santa Maria - RS; Oliveira dos Campinhos – BA; Nova Odessa – SP.

c Detected in one sample.

d ND: not detected.
